# INTERGENERATIONAL RELATIONSHIPS AMONG OLDER ADULTS AND ADULT CHILDREN: AMBIVALENT FEELINGS

**DOI:** 10.1093/geroni/igz038.638

**Published:** 2019-11-08

**Authors:** Sofia von Humboldt, Isabel Leal

**Affiliations:** William James Center for Research, ISPA - Instituto Universitario, Lisbon, Portugal

## Abstract

Objectives: The relationship of older adults with their adult children involves great emotional complexity and the quality of these relationships is associated with older adults’ well-being. This qualitative study aims to examine how older adults conceptualize intergenerational relationships with adult children. Methods: The present study on qualitative data collected from in-depth interviews was conducted with English and Portuguese older adults living in the community, designed to address their perspectives on intergenerational relations with adult children. 316 older adults participated in our study. The mean age of this group was 71.2 years. 65.3% were women, and a majority (54.7%) had a partner. Results: Content analysis generated four themes: affection and integration; satisfaction in the relationship; privacy and boundaries; financial support. Conclusions: Intergenerational relationships are experienced by older adults with ambivalence and and stress the contradictory expectations of older adults with grandchildren.

